# How Do Proactive Environmental Strategies Affect Green Innovation? The Moderating Role of Environmental Regulations and Firm Performance

**DOI:** 10.3390/ijerph18179083

**Published:** 2021-08-28

**Authors:** Naveedullah Mulaessa, Lefen Lin

**Affiliations:** College of Finance, Nanjing Agricultural University, Nanjing 210095, China

**Keywords:** proactive environmental strategies, green innovation, environmental regulations, firm performance

## Abstract

Global warming has gained the attention of researchers and authorities to work on the environmental glitches. Prior researchers highlighted that the industrial sector is more responsible for these environmental glitches. The industrial sector is highly participated for climate change problems. In the light of firm’s sustainable development goals, this study focuses on the proactive environmental strategies for green innovation. Furthermore, this study considers the link amid environmental regulations and green innovation, firm performance, and green innovation. Most importantly, this study applies the moderating role of environmental regulations and firm performance on the link amid proactive environmental strategies and green innovation. The outcomes with ordinary least square, fixed effect, generalized method of moments, and feasible generalized least square presents unique conclusions. This study concluded that firms with proactive environmental strategies are more valuable for green innovation practices. The environmental regulations promote green innovative practices. Similarly, firm performance also encourages the firm for green innovative practices. Importantly, these outcomes suggest that environmental regulations positively moderate the link amid proactive environmental strategies and green innovation. In addition, firm performance also plays positive role for positivity amid proactive environmental strategies and green innovation. These findings are imperative addition into the narrow literature of environmental practices at firm level in Pakistan. Moreover, this study suggests various guidelines and directions for policy makers, owners, governments, and stakeholders as promoting the environmental practices for higher profitability as well as minimizing industrial negative effects.

## 1. Introduction

Climate change problems leading to the global warming and it is getting worse with the passage of time therefore, institutions and government have major concern for the minimization of environmental glitches [1]. Many economies have formed various policies and regulations for controlling global warming which are being implemented by the governments or institutions [2]. The industrial sector is highly important for the economic development of a country but it also produces environmental glitches, especially firms in developing economies [3]. Hence, these environmental problems generate the need to investigate the industrial sector in the context of environmental practices. Prior literature on environmental practices provided reasons to motivate firms to seek proactive environmental strategies [4]. Moreover, currently, the sustainable environment is very important for firms because it enhances the shareholders’ confidence, as well [5].

Yet, there is still debate available about firms’ current tactics to environmental management and the requirement [6,7,8], firms’ environmental management tactics usually fall short of what is necessary for the sustainable development of the natural environment [9]. Therefore, scholars’ focus has been shifted towards the proactive environmental strategies [7] that improve the sustainable development of the natural environment. Furthermore, scholars reported that environmental regulations can play an imperative role to enhance the sustainable development [10]. The involvement of firms for managing sustainable development is not properly developed yet [9]. The role of green innovation is considered as the sustainable development of the natural environment [11].

However, various prior studies have highlighted the importance of proactive environmental strategies for green innovation [12,13,14]; however, no study has provided the reason for this positive association between proactive environmental strategies and green innovation. Therefore, this study pointed out two questions. Firstly, is proactive environmental strategies really valuable for green innovation? If yes, then what are major factors or elements which make it possible? Hence, this study proposes the role of environmental regulations and firm performance for the improvement of proactive environmental strategies and green innovation. In this context, the natural resource-based view theory stated firms with proactive environmental strategies could gain competitive advantage influenced by the firm performance [15,16]. In addition, Porter also shed light on the imperative role of environmental regulations for the improvement environmental strategies and innovative practices. Environmental regulations forces firms to create proactive environmental strategies, which leads to the green innovation. Furthermore, the greed of firm performance also encourages firms to adopt environmental strategies for innovation.

The context of developing economies provides great motivation for this study because the environment problems are severe in the firms of developing economies than the developed economy [17]. The lack of capital is another reason for less involvement of firms into the environmental practices in developing countries [18]. In addition, the environmental glitches are severe in the Asia and Pacific’s territory [19]. Moreover, the industrial sector is growing very fast in Pakistan, India, and China which also produces environmental problems [20]. There is an intense need to work on the Pakistani market because Pakistan is associated with higher level of environmental and other social problems [21]. Furthermore, Pakistan has huge level of transparency issues [22]. Despite all these problems, Pakistan has a proper legal system for social and environmental responsibility to enhance stakeholder confidence (GOP, 1983).

Hence, this study has developed various motives such as firstly investigating the impact of proactive environmental strategies on green innovation that have never been discussed before in Pakistani context. Secondly, this study examines the role of environmental regulations for green innovation and majorly the moderating role of environmental regulations for association between proactive environmental strategies and green innovation. Thirdly, this study investigates the role of firm performance for green innovation and similarly, the moderating role of firm performance for association between proactive environmental strategies and green innovation. This study first applied ordinary least square (OLS) for finding endogeneity in panel date and our results confirmed the presence of endogeneity. In addition, after the implication of fixed effect, generalized method of moments, and feasible generalized least square, the outcomes reveal the positive link between proactive environmental strategies and green innovation, environmental regulations and green innovation, and firm performance and green innovation. Most importantly, the outcomes of this study support the moderating role of environmental regulations and firm performance for a positive link between proactive environmental strategies and green innovation.

This study contributes to the narrow literature on environmental practices in Pakistan as promoting the role of proactive environmental strategies for green innovation. Furthermore, this study highlights the importance of environmental regulations and firm performance for proactive environmental strategies and green innovation. Prior to this, no scholar had provided such evidence; therefore, this is a unique study of its type. Moreover, this study theoretically contributed as extending the natural resource view-based theory and the Porter hypothesis. This study has multiple managerial implications for policy makers and other concerning authorities. This study supported those firms that are involved in environmental practices. This study also encourages the regulatory bodies to impose stricter environmental regulations for the improvement of environmental and green innovative practices.

This article features the following sections: Section 2 highlights the theoretical analysis and hypothesis construction. Section 3 explain the sample and data collection with the variable measurement. Section 4 reveals the methods, models, results, and discussion. The last section consists of conclusion, managerial implications, and limitations.

## 2. Theoretical Framework and Hypothesis Construction

The environmental strategies are of huge importance for firms because it could achieve competitive advantage and improve firm performance as well. Therefore, in this context, the natural resource based theory shed light on the firm activities when firms involve in making natural environment clean [15,16]. Generally, this theory focuses on three objectives as, environmental strategies for reducing pollution and ensuring product stewardship and sustainable development. Environmental strategies are normally considered as cost-reducing, differentiation, and hybrid [23]. Firm investing in proactive environmental strategies for reducing operational and future liability cost which can gain competitive advantage [24]. Meeting the objective of zero emissions and wastes is possible only when firms try to remove the pollutant from the production process.

Therefore, the proactive environmental strategies are supposed to be cost-reducing and environment friendly which leads to innovation [25]. Thus, by advancing the natural resource-based view theory, this study proposes that the firms could gain competitive advantage as involving in proactive environmental strategies which leads to green innovation and profitability. Hart and Dowell [16] reported that the firms with natural resource strategies could gain firm performance and improve sustainable environment as well. Researchers have previously expressed the belief that this theory is highly beneficial to measure the firm performance as considering corporate social objectives and sustainable development [26]. Green innovation is an imperative approach to measure the firm sustainable development [27].

In addition, to examine the role of environmental regulations for firm innovation we need to go back in the Porter era. Porter presented hypotheses which supported the role of environmental regulations for firm innovative practices as achieving competitive advantage [28]. Overall, Porter presented two views as first mover advantage theory and innovative compensation theory innovation [28,29,30]. These theories highlighted the importance of environmental regulations for green innovation. Firms that are involved in environmental activities can gain first mover advantage that also captures competitive advantage. This situation develops firm goodwill for the long run and automatically enhances profitability.

### 2.1. Proactive Environmental Strategies and Green Innovation

The willingness of promoting sustainable environment by firms are not formally developed yet [9]. Therefore, there is still debate available about competitive advantage by involving in proactive environmental strategies. Green innovation is supposed to be a sustainable development at firm level [11]. Green innovation is explained as the improved or new product, technology, process, and practice for removing or minimizing environmental glitches [12,13]. Zhou et al. [31] stated that the focus of researchers is shifted towards proactive environmental strategies for the improvement of sustainable performance. In this support, the researchers highlighted that the firms with environmental strategies have impressive sustainable performance than those firms that do not have it [14]. The strong business strategy is considered as firms have proactive environmental strategies [32].

Green innovation normally sheds light on the improvement of process and product which leads to environment friendly products [11]. Using appropriate raw materials, reducing waste, making products with environment friendly principles are the major part of proactive environmental strategies [33]. Generally, green innovation has positive association with a firm’s overall performance [34,35]. Therefore, firms with the help of green innovation could reduce cost and improve productivity [36]. A recent study reported the positive role of environmental strategies on environmental performance which also improve the corporate social practices [34]. The strength of proactive environmental strategies can lead to the higher green innovation [11].

The proactive environmental strategies are imperative for achieving competitive advantage through the innovation [28]. Stakeholders interested more in those firms which are environmentally conscious [37]. Therefore, proactive environmental strategies enhance the stakeholders’ confidence. Consequently, firms with proactive environmental strategies should be able to gain the innovation. At market level, firms with proactive environmental strategies are associated with first mover advantage [4]. Moreover, they concluded that sustainable development of the firms is linked with proactive environmental strategies. Proactive environmental strategies force firms to involve in eco-friendly practices for the sake of minimizing environmental glitches [24].

Many prior studies have identified a link between proactive environmental strategy and competitive advantage but their focus was limited to the green innovation [38]. Green innovation is a valuable tool to remove the negative climate impacts. In previous decades, there are huge level industrial development throughout the world but with negative industrial impacts on environment. Many researchers argue that the industrial sector has major responsibility to damage the climate [3]. Moreover, the manufacturing sector has a large degree of participation in damaging the environment because of the involvement of production process [11]. Therefore, there is a great need to probe the environmental practices in manufacturing firms. In addition, Liu et al. [39] also supported the role of proactive environmental strategies for environmental performance. Environmental legitimacy helps to improve the green innovation practices at firm level [40]. Chen et al. [41] stated that proactive strategies enhance green creativity and product development, as well. Therefore, we propose this hypothesis based on the above-mentioned arguments:

**Hypothesis** **1** **(H1).** *Proactive environmental strategies are valuable for the improvement of green innovation*.

### 2.2. The Moderating Role of Environmental Regulations

The concept of environmental regulations was introduces in the 1970s, and has since become a hot issue due to global warming [20]. Environmental regulations play a vital role in innovative firm practices [28]. Luo, Salman and Lu [27] reported that environmental regulations force companies towards green innovation. Environmental regulations invited firms for green innovation through the decreasing pollution related practices and simultaneously enhancing the production of products. Zhang, Wang and Zhao [40] demonstrated that Chinese firms used green innovation as an important strategic practice to protect the ecological life. However, the involvement of firms into green innovation practices is low and the Chinese government’s focus on this issue ought to correct this problem [42].

Regarding effect of environmental regulations on green innovation, we can refer back to [28], which reported the benefits of environmental regulations on firm innovation practices. For instance, Song et al. [3] discovered the inverted U-shape association amid environmental regulations and green innovation. Well-designed environmental regulations refer to well-made environmental standards, which form a win-win condition while making new or improved products for competitiveness. Green innovation and environmental regulations also enhance firm reputation in the market which build shareholders confidence. Firms with environmental regulations automatically produces green innovation [43]. The concept of green innovation is growing very fast in China and firms considering environmental regulations for developing firms sustainability [44]. Generally, green innovation is depends upon the type of environmental regulations and all type of environmental regulations are positively linked with green innovation [45]. Moreover, Song et al. [3] highlighted that environmental regulation is a key driver to boost up the firm green innovation practices.

Generally, environmental regulations participated in the environmental strategies which deal with the environmental objectives at the early stages of product development for reducing its negative impact [46]. Environmental regulations majorly focus on the penalties [2]. For instance, if a firm violates the environmental standards, the relevant authority may punish them and forces them to follow the environmental regulations and these are part of environmental strategies [47]. Moreover, environmental regulations enable firms to enhance the environmental protection practices to maintain goodwill in international market, thus, firms also consider proactive environmental strategies [48].

In addition, environmental regulations play the role of providing strict supervision on the proactive environmental strategies [41]. Hence, firms will further minimize the cost in environmental safety and are encouraged to develop the proper environmental strategies [49]. Further, environmental regulations incorporate both reactive and proactive environmental strategies. For example, a firm working on environmental regulations may also receive attention from governments in the form of grant or other rewards for removal of environmentally negative effects [50]. Motivation within firms for managing cost and environmental protection is an effective strategy [51].

Environmental regulations normally guide firms to implement the proactive en-vironmental strategies which also focus on environment protection while busy in making profit [52]. Market based incentives encourage firms to participate more in en-vironmental strategies as following environmental regulations [53]. In support of this, Peng et al. [54] concluded that proactive environmental strategies and environmental regulations are significantly connected. Firstly, before examining the moderating role of environmental regulations there is a need to examine the direct effect. Therefore, we propose the following hypothesis on the basis of the above theoretical evaluation and discussion as:

**Hypothesis** **2** **(H2).** *There is a positive association between environmental regulations and green innovation*.

**Hypothesis** **3** **(H3).** *Proactive environmental strategies and green innovation are positively connected with the moderating role of environmental regulations*.

### 2.3. The Moderating Role of Firm Performance

Green innovation is helpful in reducing the negative impact on the environment and enhances firm performance, as well [55]. The resource-based view theory is highly beneficial in this context because it sheds light on the link between proactive environmental strategies and firm performance [38]. Proactive environmental strategies can be linked with the development of exclusive firm characteristics [56]. Proactive environmental strategies have capabilities that permit firms to line up their strategies with the uncertain, changing, and complex business environment [57]. They are also associated with firm competitiveness which may capture firm competitive advantage [58]. Triguero et al. [59] asserted that green innovation plays a positive role in improving firm environmental and economic performance.

As prior literature reveals that proactive environmental strategies are very important for green innovation and firms with green innovation could also improve firm performance [38]. Environmental management and firm performance are linked with help of innovative practices [28]. Proactive environmental strategies necessitate firms to protect the environmental impact by using innovative approach as making eco-friendly products which ultimately boosts firm performance [26]. The greed of firm performance pushes firms to make proactive environmental strategies that take firms towards sustainable development.

Moreover, Ghisetti and Rennings [60] highlighted that profitability and competitive advantage are positively linked because of the proactive environmental strategies. Proactive environmental strategies are connected with various benefits such as saving costs, reducing environmental impact, and positive change in economic returns. In addition, numerous scholars have probed empirically the relationship between proactive environmental strategies and firm performance, and discovered the positive relationship between them [61,62]. Klassen and Whybark’s research [63] also supports the role of proactive strategies in the improvement of firm performance. Proactive environmental strategies positively change firm performance while adding organization capabilities [56,64].

**Figure 1:**
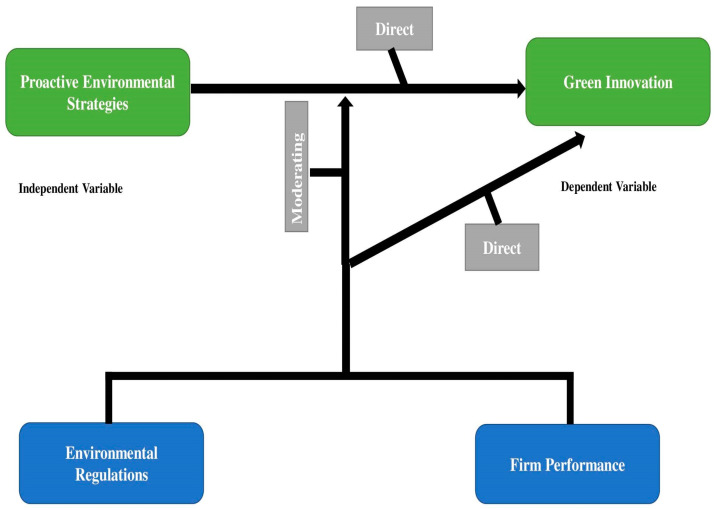
Conceptual diagram.

Firm performance is the major aim of every firm, therefore, top management uses various approaches, such as involvement with environmental practices [20]. A number of studies have shown that firms with proactive environmental strategies are supposed to follow the best business strategy [30,61,65]. Moreover, having an environmental strategy promotes environmental protection initiatives which help companies with strategic planning [66]. Firm performance generally depends on strategic planning. Figure 1 reveals the conceptual framework of this study.

This study proposes the following two hypotheses based on arguments and theoretical background.

**Hypothesis** **4** **(H4).** *There is a positive association between firm performance and green innovation*.

**Hypothesis** **5** **(H5).** *Proactiveenvironmental strategies and green innovation are positively connected with the moderating role of firm performance*.

## 3. Data and Sample Selection

This study selected Pakistani stock market and the manufacturing firms have been selected for investigation because most prior studies reported that these firms have major involvement in environmental glitches which had to be investigated [67,68]. Moreover, manufacturing firms in developing economies are considered more responsible for pollution [67]. Manufacturing firms are known for being a major source of waste, air and water pollution, and a major contributor to climate change [11]. Therefore, manufacturing firms have a greater responsibility to publish accurate figures of corporate social aspects than the other sectors [69]. We collected data from multiple sources such as Pakistan Stock Exchange (PSX), firms’ annual and sustainability reports, and the Intellectual Property Organization of Pakistan (IPO-PAK). The time span of 2009–2018 was used for the collection of data. Finally, 296 firms (2956 observation) were selected for the completion of this investigation.

### 3.1. Environmental Strategies in Pakistani Context

Pakistan is a country that is associated with multiple economic and social problems [20]. Additionally, the corruption rate in Pakistan is also higher [21,70]. The larger firms have been involved in environmental activities in Pakistan [71]. Consequently, firms in this situation produce substandard products, disrupt human integrity, and are involved in child labor practices. However, corporate social practices are supposed to be an imperative model for dealing with the above-mentioned issues and enhancing the shareholders’ confidence. Various authorities have raised the issue related to the social and environmental activities in Pakistan, for example, the Norwegian agency, the United Nations, and the Government of Pakistan (GOP) [72]. Much of the wastage of industrial material in Pakistan is caused by the manufacturing sector and it leads to low quality of water and environmental contamination glitches [73]. Despite all these problems, Pakistan has a proper legal system for solving the social and environmental issues, which enhances the confidence of stakeholders [74]. Importantly, the Pakistani government has recently implemented a system, namely, environmental management, that is linked with the National Environmental Quality Standards of Pakistan (NEQS), to deal with climate change challenges.

The GOP have been involved to an extreme level in reducing industrial negative impacts, thus, they have applied multiple social and environmental policies [20]. In addition, the Pakistani market has special ordinance related to environmental practices, namely, Environmental Protection Ordinance (EPO) [74]. This ordinance focuses primarily on the social and environmental practices of industrial sector for removing negative impact. Moreover, the Pakistani market has taken imperative initiatives as developing an act related to environmental protection Pakistan Environmental Protection Act (PEPA) in 1997 for more firming up the environmental actions [75]. This act clearly presented instructions to the industrial sector for participation in social and environmental practices and disclosing them. The GOP think that these rules and regulations are imperative to raise the shareholders’ confidence. Additionally, these environmental rules are much identical with other developing economies as well, for example, Egypt, Sri-Lanka, India, and Tunisia, had also developed the same regulations for controlling industrial negative impacts [76,77].

The Securities and Exchange Commission of Pakistan (SECP) is a major body in Pakistan for monitoring and controlling the industrial sector [78]. The SECP has developed various rules and regulations which require of the firms to be involved in cleaner production practices. In addition, the Pakistani market also follows international quality standards ISO: 9000; 9001, ISO: 18001, ISO: 14000; 14001, which are being implemented by the SECP [79]. The GOP is always looking for means to control these industrial negative effects and has formed another sustainable strategies, namely, “Draft Hazardous Waste and Hazardous Substances Rules” [80].

### 3.2. Variable Construction

#### 3.2.1. Dependent Variable

Green innovation (GI) is our dependent variable and prior research has stated that the accurate measurement of the variables yields effective results in empirical studies [20]. Generally, firms invested in patents are considered to be involved in green innovation [43]. Therefore, we used environment patent applications applied by firms as green innovation [43,81]. We normally selected those firms which have invested in patens for green innovation (GI) which includes keywords such as clean, green, cycle, sustainable, ecological, saving, low carbon, environmental protection, and reduction of environmental pollution and emissions [82,83]. Generally, firms invest in patents to maintain their social image and technological benefits and thus to make greater profits. Therefore, the patent applications are supposed to be the best tool to measure a firm’s innovative practices [43]. Therefore, we measure the green innovation with the number of patents by firms during the period [84,85]. Hence, the data for patent applications were collected by the Intellectual Property Organization of Pakistan (IPO-PAK).

#### 3.2.2. Independent Variable

This study used proactive environmental strategies (PES) as an independent variable. This study constructs PES proxy as the firm’s total investment in research and development section [86]. Prior scholars also supported this proxy for the measurement of PES [4,87].

#### 3.2.3. Moderating Variables

This study used two moderating variables—environmental regulations and firm performance—which were first used as independent variables to examine their direct impact. First, we used environmental regulations (ER) because environmental regulations have gained a great deal of attention throughout the world due to environmental problems. We determine the environmental regulations as total fees paid by the firms for controlling of pollution divided by the firm’s output value [20,88,89]. Secondly, this study employed the Sustainable Growth Rate (SGR) as another external firm performance mean. The current trend has changed because stakeholders mostly believe in social contribution and development with the motive of profit. SGR reveals firm financial and social policies for the enhancement of sales [90]. Furthermore, SGR participates in the enhancement of firm sales and revenue without enhancing leverage. Therefore, by following Feng et al. [91] we have determined SGR as the combination of profit margin, dividend payout ratio, total debt and equity ratio, and total asset to total sales.

#### 3.2.4. Control Variables

A number of control variables have been used in this study because these variables play a supportive role, especially in the empirical studies [92]. Firstly, we used firm size and determined it by taking the natural log of total assets [93]. Secondly, leverage was determined as the ratio of total liabilities to total assets [91]. Thirdly, the ratio of plant, property, and equipment is calculated from total sales [94]. Fourthly, the asset turnover ratio is determined as the total sales to total assets [95]. Lastly, environmental awareness is calculated as, total investment made by a firm for landscaping and divided by the number of employees [96].

## 4. Methods

### 4.1. Endogeneity Test

This study was conducted on panel data and these data generally carry endogeneity problems [97]. Endogeneity problems are defined as occurring when explanatory variables and error terms develop correlations during regression, which may produce biased and unreliable results [98]. In addition, two more reasons are available for endogeneity problem such as, firstly, the occurrence of causality among variables and secondly, dependent and independent variables not having a direct effect while other variables have correlations between them [93]. Furthermore, imprecise inferences and contradictory estimates are major causes of endogeneity bias that may reveal uncertain results and inappropriate theoretical clarification. In this order, prior to examining the final results, there is a pronounced need to conduct the endogeneity test on panel data, which many previous scholars have missed in their studies. Hence, this study calculates the endogeneity by using Ordinary Least Square (OLS) regression and the Wald test [99,100].

For the estimation of endogeneity, we calculated the residuals of each independent variable by using ordinary least squares. Table 1 reports that the residual values’ significance level, residual of proactive environmental strategies (RESID_PES), is 0.051 (*p*-value = 1%), residual of environmental regulations (RESID_ER) is 0.251 (*p*-value = 1%), residual of firm performance (RESID_SGR) is 0.024 (*p*-value = 1%), residual of interaction between proactive environmental strategies and environmental regulations (RESID_PESER) is 3.275 (*p*-value = 1%), and residual of interaction between proactive environmental strategies and firm performance (RESID_PESSGR) is 2.055 (*p*-value = 1%). Thus, these significant values confirmed that our independent variables are endogenous. For improved validity of the results, we conducted the Wald test and its significance level also confirms the endogeneity in our panel data (Semykina and Wooldridge, 2010, Kim and Kim, 2011). These results suggested that there is a need for appropriate technique to remove or cover the endogeneity for valid outcomes.

### 4.2. Fixed Effect and Generalized Method of Moments

This study used the panel data of Pakistani manufacturing firms, and prior researchers reported that panel data are associated with endogeneity and heteroscedasticity issues [20,101]. Therefore, we carefully selected statistical methods to deal with similar issues in our data set. Firstly, a fixed-effect model was employed to cover the inaudible heterogeneity based on the Hausman test [102,103]. The Hausman results permitted us to employ a fixed-effect model instead of a random effect model. Secondly, this study used the generalized method of moments (GMM) for solving the endogeneity issues. Researchers previously strongly believed that the use of generalized method of moments is the most suitable approach for correcting endogeneity compared to other methods [20,91,104]. Finally, this study employed the feasible generalized least square (FGLS) model as a robustness test based on the Hausman test to investigate the heteroscedasticity and autocorrelation from panel data [105,106,107,108].

### 4.3. Models

For testing of hypothesis 1 to 5 we have constructed the following equations:

(1) $$GI_{i,t}=\alpha_{1}+\beta_{1}PES_{1i,t}+\gamma_{1}Z_{i,t}+\mu_{i,t}$$

(2) $$GI_{i,t}=\alpha_{2}+\beta_{2}ER_{2i,t}+\gamma_{2}Z_{i,t}+\mu_{i,t}$$

(3) $$GI_{i,t}=\alpha_{3}+\beta_{3}SGR_{3i,t}+\gamma_{3}Z_{i,t}+\mu_{i,t}$$

(4) $$GI_{i,t}=\alpha_{4}+\beta_{4}PES_{4i,t}+\beta_{5}ER_{5i,t}+\beta_{6}PES*ER_{6i,t}+\gamma_{4}Z_{i,t}+\mu_{i,t}$$

(5) $$GI_{i,t}=\alpha_{5}+\beta_{7}PES_{7i,t}+\beta_{8}SGR_{8i,t}+\beta_{9}PES*SGR_{9i,t}+\gamma_{5}Z_{i,t}+\mu_{i,t}$$

From Equation (5), $GI_{i,t}$ represents green innovation of firms *i* at year *t*; *PES*—proactive environmental strategies; $ER_{1i,t}$—environmental regulations; $SGR_{1i,t}$—shows firm performance; *PES*∗*ER*—the interaction between proactive environmental strategies and environmental regulations; *PES*∗*SGR*—the interaction amid proactive environmental strategies and firm performance firms *i* at year *t*$;Z_{i,t}$—control variables of firm *i* at year *t*; $\mu_{i,t}$—error term; $\alpha_{n}$—constant term, *n* = 1; $\beta_{m},\;\gamma_{n}$—Coefficients to be estimated; *m* = 1, 2, 3, 4, 5, 6, 7, 8, 9.

### 4.4. Results

Prior to testing the hypotheses, we performed descriptive statistics and a correlations test, which are reported in Table 2. The mean and standard deviation values of all variables are reported in this table; moreover, this table indicates the correlations between all variables. There is positive and significant association between all variables. Table 3 indicates the various models and reveals the results of association between proactive environmental strategies (PES) and green innovation (GI), environmental regulations (ER) and green innovation (GI), firm performance (SGR) and green innovation (GI) with both fixed effect and generalized method of moments model. There is positive and significant connection discovered between proactive environmental strategies (PES) and green innovation (GI), as model 1 shows (β_ = 0.027, *p* = 0.05, β_ = 0.049, *p* = 0.05). Proactive environmental strategies play a significant role in green innovation. In addition, Table 3 highlights the results of association between environmental regulations (ER) and green innovation (GI), as shown in model 2 (β_ = 0.151, *p* = 0.01, β_ = 0.099, *p* = 0.01). These outcomes confirmed that having environmental regulations leads to green innovation for firms. Furthermore, Table 3 reveals the results of connection between firm performance (SGR) and green innovation (GI), as presented in model 3 (β_ = 0.070, *p* = 0.01, β_ = 0.086, *p* = 0.01). Thus, these results supported the role of firm performance in improving green innovation practices. Table 3 also presents the Hausman test results (β_ = 58.91, *p* = 0.01, β_ = 104.53, *p* = 0.01, and β_ = 100.55, *p* = 0.01). Therefore, these results supported the implication of the fixed effect model rather the random effect model.

Table 4 indicates the results of moderating effects such as model 4 and 5. The moderating role of environmental regulations on the association between proactive environmental strategies and green innovation is reported as the interaction of proactive environmental strategies and environmental regulations (PES*ER) in model 4 (β_ = 2.192, *p* = 0.01, β_ = 0.657, *p* = 0.01). Thus, these empirical outcomes confirmed that environmental regulations are highly important for controlling proactive environmental strategies which lead to green innovation. Moreover, Table 4 reveals the results on the moderating role of firm performance on association between proactive environmental strategies and green innovation reported as the interaction of proactive environmental strategies and environmental regulations (PES*ER) in model 5 (β_ = 1.953, *p* = 0.01, β_ = 1.996, *p* = 0.01). These results also highlighted the importance of firm performance for proactive environmental strategies and green innovation. In addition, for supporting the implication of the fixed effect model, the Hausman test values of model 4 and model 5 were calculated (β_ = 430.99, *p* = 0.01, β_ = 69.19, *p* = 0.01).

### 4.5. Additional Test

For improved accuracy of the results, this study employed the feasible generalized least squares (FGLS) as a robustness test. Table 5 indicates 5 models which shed light on the association between proactive environmental strategies (PES) and green innovation (GI), environmental regulations (ER) and green innovation (GI), firm performance (SGR) and green innovation (GI) and the moderating results. Model 1 reveals proactive environmental strategies (PES) with green innovation (GI) (β_ = 0.021, *p* = 0.01), Model 2 shows environmental regulations (ER) with green innovation (GI) (β_ = 0.148, *p* = 0.01), Model 3 presents firm performance (SGR) with green innovation (GI) (β_ = 0.019, *p* = 0.01). Similarly, models 4 and 5 indicate the moderating results as interaction between proactive environmental strategies and environmental regulations PESER (β_ = 4.074, *p* = 0.01) and interaction between proactive environmental strategies and firm performance PESSGR (β_ = 1.863, *p* = 0.01). Hence, all these results also confirm all our hypotheses.

### 4.6. Discussion

The industrial sector is highly important for the development and growth of a country but it also leads to various environmental issues. Environmental glitches, especially for the industrial sector, have attracted much attention in developing countries because this sector has a greater involvement in creating environmental problems [20]. Presently, various economies are facing environmental glitches that have huge effects on life of human beings and the climate; therefore, institutions and governments are strongly focusing on these issues. In this context, this study suggested the importance of proactive environmental strategies for green innovation. Investigating the first hypothesis confirmed that green innovation could be enhanced with proactive environmental strategies. Natural resource-based theory contends that the participation of firms into environmental practices produces green innovation [15,16]. Proactive environmental strategies create a firm positive image in the market and also reduce wastage during production [23]. Furthermore, the greed of competitive advantage compels firms to adopt proactive environmental strategies [24] also leads firms to become more involved in green innovation [16]. In this era, green innovation or sustainable development are the key to success for firms and therefore firms develop proactive environmental strategies [14].

Prior literature shows the positive role of proactive environmental strategies for green innovation; however, no studies had presented the reason for this positive relationship. Therefore, our study proposed the role of environmental regulations and firm performance for checking the relationship between proactive environmental strategies and green innovation. Prior to checking the moderating effects, there is a need to examine the direct effect of these moderating variables first. The environmental regulations also play a positive role in a firm’s green innovative practices [28]. Hence, the results of our second hypothesis also stated that environmental regulation is a valuable approach to make firms engage in green innovation. Environmental regulations have an imperative role in firm innovation and Porter [28] provided support for these relationships in 1970. Environmental regulations push firms towards innovative practices which ultimately enhances firm goodwill [27]. The firms use environmental regulations for removing industrial negative effects and green innovative objectives [40]. Researchers believed that firms with environmental regulations win the confidence of shareholders and produce green innovation [43].

However, after checking the direct effect of environmental regulations on green innovation, this study proposes the moderating role of environmental regulations on the association between proactive environmental strategies and green innovation. The results regarding our third hypothesis reported that proactive environmental strategies and green innovation are positively connected because of the moderating role of environmental regulations. Theoretically, both of Porter’s theories, first mover advantage and innovative compensation, supported the role of environmental regulations for proactive environmental strategies and green innovation [28,29,30]. Environmental regulations encourage firms to make proactive environmental strategies which also capture the firm’s sustainable development [46]. Moreover, the pressure of penalty is also another reason for engaging in proactive environmental strategies [2]. The international reputation of firms also encourages green innovation with the help of proactive environmental strategies and environmental regulations [48]. Environmental regulations apply stricter requirements on firms for adopting proactive environmental strategies [41]. Saving costs is also another motivation for firms to become involved in proactive environmental strategies following green innovation [49]. Generally, cost saving and reducing industrial negative impact with the help of environmental practices is an imperative business strategy [51].

This study also focuses on the role of firm performance in green innovation. Green innovation ultimately captures higher profits for a firm [55]. Thus, the results of our fourth hypothesis concluded that firm performance and green innovation are positively linked. Theoretically, the resource-based theory encouraged firms to engage in green innovation practices which also leads to higher profit [38]. Green innovation also captures competitive advantage which ultimately improves firm performance [58]. Sustainable development makes firm innovative with the improvement of environmental and economic performance [59]. Porter also stated that firm performance could be enhanced with a firm’s innovative practices [28]. Green innovation involves firms in making new or improved products which have a positive impact on firm performance [26].

Lastly, our fifth hypothesis discovered that proactive environmental strategies and green innovation have a positive relationship with the moderating role of firm performance. Every firm is looking for means to maximize the profit so proactive environmental strategies are supposed to be the best approach for enhancing profitability [60]. The struggle of firm performance lays in making innovation at firm level with the help of environmental strategies [61,62]. Proactive environmental strategies enhance firm capabilities which automatically increases firm profit [56,64].

## 5. Conclusions and Managerial Implications

Environmental issues change the climate which leads to global warming. Therefore, the pressure of sustainable development and eco-environment increases day by day, and this motivates governments and authorities to work in this domain. The involvement of firms in environmental practices not only enhances a firm’s reputation as a socially responsible firm but also leads to gaining the first mover advantage. In this context, we selected 296 Pakistani manufacturing firms (2956 observation) for the period of 2009–2018. For analysis, we applied various econometric approaches, such as ordinary least squares, first applied to probe the endogeneity in our data. After that fixed effect model, the generalized method of moments was also applied. Moreover, for robustness, the feasible generalized least squares was also applied. Our results reported notable outcomes, such as, firstly, that the practices of green innovation can be enhanced with the proper proactive environmental strategies. This study discovered that there is a positive link between proactive environmental strategies and green innovation. Secondly, environmental regulations also play a positive role for the improvement of green innovation. Our results stated that environmental regulations are positively linked with green innovation. Thirdly, green innovation can also be improved with the greed of firm performance. This study found that there is a positive association between green innovation and firm performance. Most importantly, our results suggested that proactive environmental strategies and green innovation are positively linked with the moderating role of environmental regulations. Similarly, the study reveals that proactive environmental strategies and green innovation are positively linked with the moderating role of firm performance. Finally, we can say that this study promoted environmental practices for the betterment of environmental problems and firm profit.

### 5.1. Managerial Implications

The outcomes of this study suggest multiple implications for policy makers, managers, governments, and institutions. This study highlights the importance of environmental strategies for improving of innovative practices. This study shed light on the imperative role of environmental regulations for improving the proactive environmental strategies and green innovation. Moreover, this study motivates top management to become involved environmental activities. Firms which introduced proactive environmental strategies are supposed to have first mover advantage. This study promotes the role of green innovation in the context of a developing economy like Pakistan where knowledge of green innovation is limited. Environmental problems could also be minimized with the help of environmental practices. This study pushes firms towards sustainable development which ultimately captures greater profit.

Firm profit also has importance in environmental strategies, so higher profits encourage firms to follow innovative strategies. This study also pushes governments and stakeholders to put pressure on firms to become involved in environmental practices. As the large shareholders are looking for long-run profit, firms with environmental practices gain reputation in the market which captures long-run profit. This study majorly shed light on the role of environmental regulations because stricter regulations compel firms to make proactive environmental strategies which automatically enhances a firm’s green innovation practice.

The SECP is a major regulatory authority in Pakistan for firms, and this study gives directions for making more innovation through environmental practices. In addition, institutions and governments should provide rewards or incentives to those firms that are following environmental practices. Moreover, the findings of this study suggest that the cost of adopting environmental practices is very low compared to the advantages. It is a great opportunity for the firms of developing economies to build a reputation in the international market as following environmental practices. This study also encourages the firms to minimize the wastage during the production process for innovation. The industrial sector can play a positive role in making the environment cleaner and positively change the climate. Additionally, the international standards play an imperative role for firms to be involved in environmental practices; thus, firms should have more focus on these standards.

### 5.2. Limitation and Future Directions

This study highlighted that large firms are much involved in environmental practices, thus, small firms should also participate in environmental activities. Moreover, this study was conducted on firms in the manufacturing sector; thus, other sectors can also be investigated. There is limited time period selected for this study because of data availability. For future investigation, this study proposes the role of a firm’s top management in creating proactive environmental strategies and green innovation.

## Figures and Tables

**Table 1:** Endogeneity results.

Independent Variables	OLS				
	Model 1	Model 2	Model 3	Model 4	Model 5
RES_PES	0.051 ***				
RES_ER		0.251 ***			
RES_SGR			0.024 ***		
RES_PESER				3.275 ***	
RES_PESSGR					2.055 ***
FS	0.957 ***	0.958 ***	0.957 **	0.96 ***	0.97 ***
LEV	−0.029 *	−0.030 *	−0.02 *	−0.028 *	−0.03 *
PPE	0.589 **	0.590 ***	0.60 ***	0.59 ***	0.55 ***
ATO	0.006 ***	0.005 ***	0.001 ***	0.005 ***	0.004 ***
EA	−0.272 ***	−0.271 ***	−0.28 ***	−0.272 ***	−0.275 ***
Constant	0.025 ***	0.024 ***	0.031 **	0.024 ***	0.026 ***
R^2^	0.6795	0.6782	0.6747	0.7532	0.7754
F-statistics	57.64 ***	45.81 ***	13.99 ***	955.36 ***	1342.37 ***
Wald Test t-stat	7.60 ***	6.77 ***	3.75 ***	30.91 ***	36.64 ***

*** 1% significance level, ** 5% significance level, * 10% significance level.

**Table 2:** Descriptive statistics and correlations.

Variables	M	SD	1	2	3	4	5	6	7	8	9	10	11
1. GI	0.31	0.39	1										
2. PES	1.29	0.79	0.21 ***	1									
3. ER	1.03	0.12	0.24 ***	−0.09 ***	1								
4. SGR	1.93	0.68	0.01	−0.53 ***	−0.04 ***	1							
5. PESER	1.25	0.44	0.75 ***	0.28 ***	0.32 ***	−0.12 ***	1						
6. PESSGR	0.04	0.08	0.78 ***	0.28 ***	0.11 ***	0.10 ***	0.73 ***	1					
7. FS	0.08	0.09	0.14 ***	0.51 ***	−0.07 ***	−0.02 ***	0.11 ***	0.22 ***	1				
8. LEV	0.23	0.40	0.54 ***	0.18 ***	0.15 ***	0.08 ***	0.45 ***	0.52 ***	0.47 ***	1			
9. PPE	0.50	0.53	0.76 ***	0.20 ***	0.22 ***	0.02 ***	0.64 ***	0.65 ***	0.10 ***	0.68 ***	1		
10. ATO	6.35	5.14	0.22 ***	0.14 ***	0.06 ***	−0.25 ***	0.19 ***	0.09 ***	−0.05 ***	−0.01 ***	0.11 ***	1	
11. EA	0.44	0.44	−0.10 ***	0.41 ***	−0.04 ***	0.07 ***	−0.01 ***	0.04 ***	0.42 ***	0.23 ***	0.18 ***	−0.25 ***	1

*** 1% significance level.

**Table 3:** Results of proactive environmental strategies, environmental regulations, and firm performance with green innovation.

	Model 1		Model 2		Model 3	
Dependent Variables Independent Variables	GI		GI		GI	
	FE	GMM	FE	GMM	FE	GMM
PES	0.027 **	0.049 **				
ER			0.151 ***	0.099 ***		
SGR					0.070 ***	0.086 ***
FS	0.092	0.351 ***	0.159 *	0.469 ***	0.138	0.433 ***
LEV	0.024	−0.008	0.022	−0.011	0.021	−0.011
PPE	0.543 ***	0.574 ***	0.539 ***	0.572 ***	0.542 ***	0.573 ***
ATO	0.007 ***	0.004 **	0.006 ***	0.004 **	0.007 ***	0.003 ***
EA	−0.463 ***	−0.605 ***	−0.457 ***	−0.602 ***	−0.460 ***	−0.603 ***
Constant	0.133 ***	0.126 ***	0.173 ***	0.177 ***	0.160 ***	0.159 ***
R^2^	0.6631		0.6651		0.6739	
F	12.11 ***		12.31 ***		13.47 ***	
N	2956	2363	2956	2363	2956	2363
Hausman Test	58.91 ***		104.53 ***		100.55 ***	
Wald Chi^2^		4072.78 ***		4074.30 ***		4071.58 ***

*** 1% significance level, ** 5% significance level, * 10% significance level.

**Table 4:** The moderating results of environmental regulations and firm performance.

	Model 4		Model 5	
Dependent Variables Independent Variables	GI		GI	
	FE	GMM	FE	GMM
PES	0.018	0.029	−0.033 **	−0.051 ***
ER	−0.019	−0.027		
PESER	2.192 ***	0.657 ***		
SGR			0.011	−0.034
PESSGR			1.953 ***	1.996 ***
FS	0.121	0.369 ***	0.019	0.222 **
LEV	0.018	−0.009	0.004	−0.024 *
PPE	0.454 ***	0.502 ***	0.399 ***	0.416 ***
ATO	0.004 ***	0.002	0.003 ***	0.001
EA	−0.385 ***	−0.531 ***	−0.341 ***	−0.455 ***
Constant	0.129 ***	0.128 ***	0.171 ***	0.223 ***
R^2^	0.7126		0.7501	
F	10.04 ***		10.87 ***	
N	2956	2363	2956	2369
Hausman Test	430.99 ***		69.19 ***	
Wald Chi^2^		4739.91 ***		6418.99 ***

*** 1% significance level, ** 5% significance level, * 10% significance level.

**Table 5:** The robustness results of feasible generalized least squares (FGLS).

	Model 1	Model 2	Model 3	Model 4	Model 5
Dependent Variables Independent Variables	GI	GI	GI	GI	GI
	FGLS	FGLS	FGLS	FGLS	FGLS
PES	0.021 ***			−0.013 ***	0.018 ***
ER		0.148 ***		0.002	
SGR			0.019 ***		0.007 *
PESER				4.074 ***	
PESSGR					1.863 ***
FS	0.630 ***	0.747 ***	0.699 ***	0.655 ***	0.319 ***
LEV	0.022	0.007	0.021	−0.033 ***	−0.001
PPE	0.579 ***	0.577 ***	0.578 ***	0.367 ***	0.388 ***
ATO	0.001 ***	0.003 **	0.004 ***	0.001 ***	0.004 ***
EA	−0.245 ***	−0.229 ***	−0.234 ***	−0.169 ***	−0.184 ***
Constant	0.013 ***	0.017 ***	0.013 ***	0.044 ***	0.016 ***
N	2956	2956	2956	2956	2956
Wald Chi^2^	20,731.67 ***	26,206.40 ***	28,329.37 ***	44,708.37 ***	38,461.68 ***

*** 1% significance level, ** 5% significance level, * 10% significance level.

## Data Availability

Not Applicable.
